# Sludge Fiber Waste and Kraft Lignin Powder as Fillers in Polylactic Acid Biocomposites: Physical, Mechanical, and Thermal Properties

**DOI:** 10.3390/polym13050672

**Published:** 2021-02-24

**Authors:** Thiago Souza da Rosa, Rosilani Trianoski, Franck Michaud, Fábio Yamashita, Setsuo Iwakiri

**Affiliations:** 1 Programa de Pós-Graduação em Engenharia Florestal, Universidade Federal do Paraná, 632, Av. Prefeito Lothário Meissner, Curitiba 80210-170, Brazil; 2 Departamento de Engenharia e Tecnologia Florestal, Universidade Federal do Paraná, 632, Av. Prefeito Lothário Meissner, Curitiba 80210-170, Brazil; rosillani@gmail.com (R.T.); setsuo.ufpr@gmail.com (S.I.); 3 LIMBHA, Ecole Supérieure du Bois, 7, Rue Christian Pauc, 44000 Nantes, France; franck.michaud@esb-campus.fr; 4 Departamento de Ciência e Tecnologia de Alimentos, Universidade Estadual de Londrina, Rodovia Celso Garcia Cid—Pr 445 Km 380, Londrina 86057-970, Brazil; fabioy@uel.br

**Keywords:** bio-based polymers, PLA, by-products, value-added products

## Abstract

In this investigation, sludge fibre waste (SFW) and Kraft lignin powder (KLP) are introduced into polylactic acid (PLA) matrix biocomposites. These alternative materials allow for both the reuse of fibre waste from paper mill sludge and a reduction in the amount of high-cost biopolymer used in the same volume. Proportions from 10 to 40 wt.% of SFW with the addition of 2.5% and 5% of KLP are incorporated in PLA by extrusion and injection moulding. The thermogravimetric properties, water absorption, tensile and flexural properties, and morphology of the fabricated biocomposites were investigated. According to the results, KLP contributes to thermically stabilising the loss resulting from the incorporation of SFW. Flexural and tensile tests reveal a more pronounced decrease in strength with an SFW ratio above 10%. The modulus of elasticity increases significantly with an SFW ratio above 20%. The strength properties are stabilised with the addition of 5% KLP. The addition of KLP presents a tendency to reduce water absorption obtained by the incorporation of SFW into biocomposites. Scanning electron micrographs evidence that KLP improves the interfacial adhesion by reducing the voids between fibres and PLA.

## 1. Introduction

The generation of solid waste from paper mill sludge is increasing and it is estimated that the global waste production ranges between 0.5 and 1.3 million tons per year [1]. Around 25% of waste from paper mills stored in sludges is intended for landfilling, land spreading, and composting [2,3,4]. In general, sludge wastes are considered non-hazardous wastes that show high alkalinity and are basically composed of organic matter, mainly cellulose fibers, and inorganic compounds, such as kaolin, talc, and calcium carbonate [3,5,6].

With regards to safety and the environment, the sludge waste from paper mills can be reused with the generation of new higher value-added products, which reduces landfill disposal [7]. Recycling sludge waste is still a challenge, but can bring several benefits and opportunities with new application solutions [8]. Indeed, different types of composite materials are being generated by fibers from sludge fiber waste (SFW), which are lightweight, durable, non-abrasive, renewable, biodegradable, and recyclable products, allowing competition with products, mainly crude-oil derivative-based with less availability or environmental disadvantages [9].

The incorporation of short natural fibers as reinforcements or fillers, such as those present in sludges, is being commonly applied in thermoplastic composites as a natural alternative to improve the mechanical and thermal properties of the products while still providing low costs and density [10]. Furthermore, the mineral components present in sludges have been used as fillers in thermoplastic products, mainly polylactic acid (PLA)-based composites with different or various loadings, thereby reducing the production costs and speeding up PLA crystallization [11].

PLA has become a sustainable alternative for replacing commodity polymers because it presents some advantages over polymers derived from petroleum, such as the low emission of greenhouse gases and a small amount of energy usage, excellent melt processability, low flammability, and renewability, especially where biodegradability is required [12,13,14,15,16,17]. However, PLA shows some disadvantages, including high costs, low toughness, low ductility, and poor thermal stability [18,19]. Although PLA is considered to be strongly hydrophobic [14,20,21], it has a certain degree of affinity with water due to the ester, oxirane, and hydroxyl groups present in its composition [22], which may undergo hydrolytic degradation in the presence of water [23].

Combining SFW and PLA biopolymers is a good alternative to address the discussed challenges. Compatibility, coupling, and adhesion quality remain key points to benefit from a composite’s raw materials. One way to improve the limitations of PLA is the incorporation of fillers, such as lignin, which allows us to improve properties while maintaining the objective of achieving bioresource materials. Lignin has been studied and added in composites due to the potential interaction between the hydroxyl and carboxyl groups of lignin and PLA. [24]. Therefore, lignin has been investigated as a biocompatibilizer between natural fibers and polymer matrices, thereby improving interfacial adhesion when used up to a specific ratio [25,26,27].

In the current investigation, SFW and kraft lignin powder (KLP) from a pulp and paper mill are used to fabricate PLA matrix biocomposites. The effect of the SFW and KLP ratio on the mechanical, physical, and thermal properties of biocomposites is investigated. The adhesion quality between the PLA and SFW is examined and investigated under scanning electron micrographs on a tensile breaking point of biocomposites.

## 2. Materials and Methods

### 2.1. Materials

The biopolymer PLA 3052D with a melt flow rate of 14 g/10 min (210 °C and 2.16 kg) and a density of 1.24 g·cm^−3^ was purchased from Nature Works LLC (Minnetonka, MN, USA). The SFW and KLP were supplied from a paper mill located in Sao Paulo, Brazil, obtained from kraft process sludge (chips from *Eucalyptus* sp.).

SFW was dried in an oven at 103 °C for 72 h and then ground in a Wiley mill to pass a 32-mesh (0.50 mm) screen in order to obtain a thin homogeneous material. The amount and composition of inorganic matter content in SFW was previously determined in Rosa et al. [28].

### 2.2. Processing of Biocomposites

Biocomposites were fabricated using a counter-rotating and interpenetrating twin-screw extruder (D-20—BGM, Sao Paulo, Brazil) with a diameter of 20 mm, L/D ratio of 34, speed of 120 rpm, and 5 heating zones with a temperature profile from 100 °C at the feeding zone to 200 °C at the die nozzle. The PLA pellets were vacuum dried at 50 ± 2 °C for 72 h and the SFW and KLP were dried at 103 ± 2 °C to constant mass before extrusion. The materials were mixed manually before extrusion by weight proportion according to the experimental design (Table 1) by adding KLP firstly into the SFW and finally the mixture into PLA. The materials were blended through an extrusion process in order to prepare biocomposite pellets suitable for injection molding. The injection molding was performed by a bench injector (AX16III—AX Plásticos, Sao Paulo, Brazil) with a volume, velocity, and fill time injection of 22 cm^3^, 30 cm^3^ s^−^¹, and 5.5 s, respectively. The barrel and mold temperature of the injection molding machine were set at 200 and 25 °C, respectively. The injection-molded specimens were obtained according to standard ASTM D638-14 [29] type IV (115 × 19 × 6 × 4 mm, for length overall, width overall, width of narrow section, and thickness, respectively) for tensile tests conjugated with a bar for flexural tests (115 × 12.5 × 4 mm for length, width, and thickness, respectively). The specimens were used for evaluation of the physical, mechanical, and thermal properties of the biocomposites.

### 2.3. Thermal Characterization

The thermal properties (thermogravimetric and its derivatives) of the SFW, KLP, PLA (neat PLA) and the developed biocomposites were investigated using simultaneous thermal analyzer (STA 449 F3 Jupiter—Netzsch, Selb, Germany). Samples of ~5 mg were heated in Al_2_O_3_ crucibles at a rate of 10 °C/min in the range of 20–800 °C under a nitrogen flow of 50 mL/min.

### 2.4. Mechanical Characterization

The mechanical properties of the developed biocomposites were evaluated. Tensile tests were performed in a computerized universal testing machine (DL 20000—EMIC/INSTRON, São Jose dos Pinhais, Brazil), with a load cell of 20 kN and a test speed of 5 mm/min^−1^, according to ASTM D638-14 [29]. The flexural tests were conducted at a crosshead speed of 1 mm/min^−1^ and a span length of 56 mm, according to ASTM D790-17 [30].

### 2.5. Water Absorption

For water absorption tests, the specimens were vacuum dried VT-5042 EK—Heraeus, Hanau, Germany) at 50 ± 2 °C for 24 h and then the dry weight was measured. The samples were dipped into a glass beaker containing distilled water at 23 ± 1 °C. The weight of the immersed specimens was measured after 2, 24, 168, and 504 h (constant weight/saturation), according to ASTM D570-98 [31].

### 2.6. Morphological Characterization

The breaking point surfaces of the tensile test samples were metalized with gold for grounding and analyzed using scanning electron microscopy (SEM) with a Tescan Vega 3 LMU (Brun, Czech) under a low vacuum (30 Pa) with an ion beam power of 15 kV.

### 2.7. Statistical Analysis

At least ten test specimens were tested for a total of three batches of each composition. To assess the effect of incorporating SFW and KLP into PLA biocomposites, the data from the physical and mechanical tests were subjected to statistical analysis using multivariate tests. The hypothesis was tested that the compositions have no significant effect on these properties through multivariate analysis of variance (MANOVA), in which the Wilks test was evaluated. This analysis was applied because the variables are correlated. Tukey’s post hoc test was applied to identify groups of means for each variable. The analyses were performed at the 5% level of significance.

## 3. Results

### 3.1. Biocomposite Characteristics

**Figure 1:**
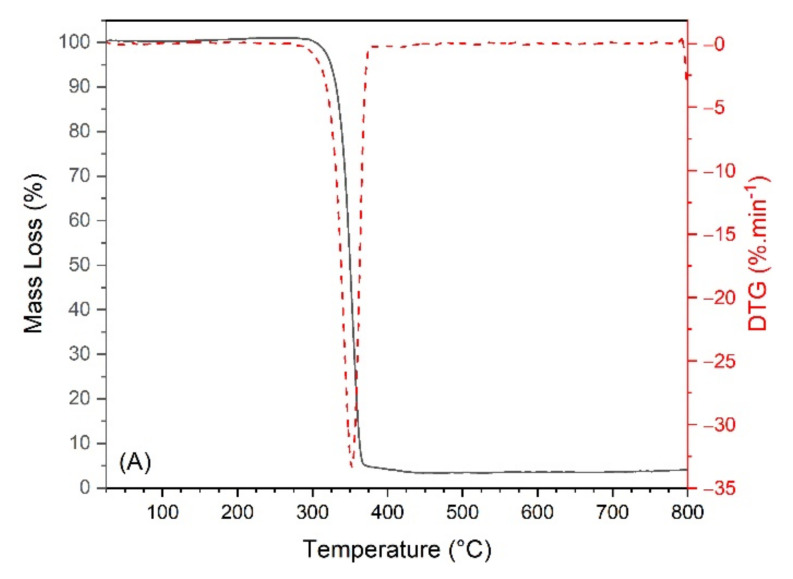

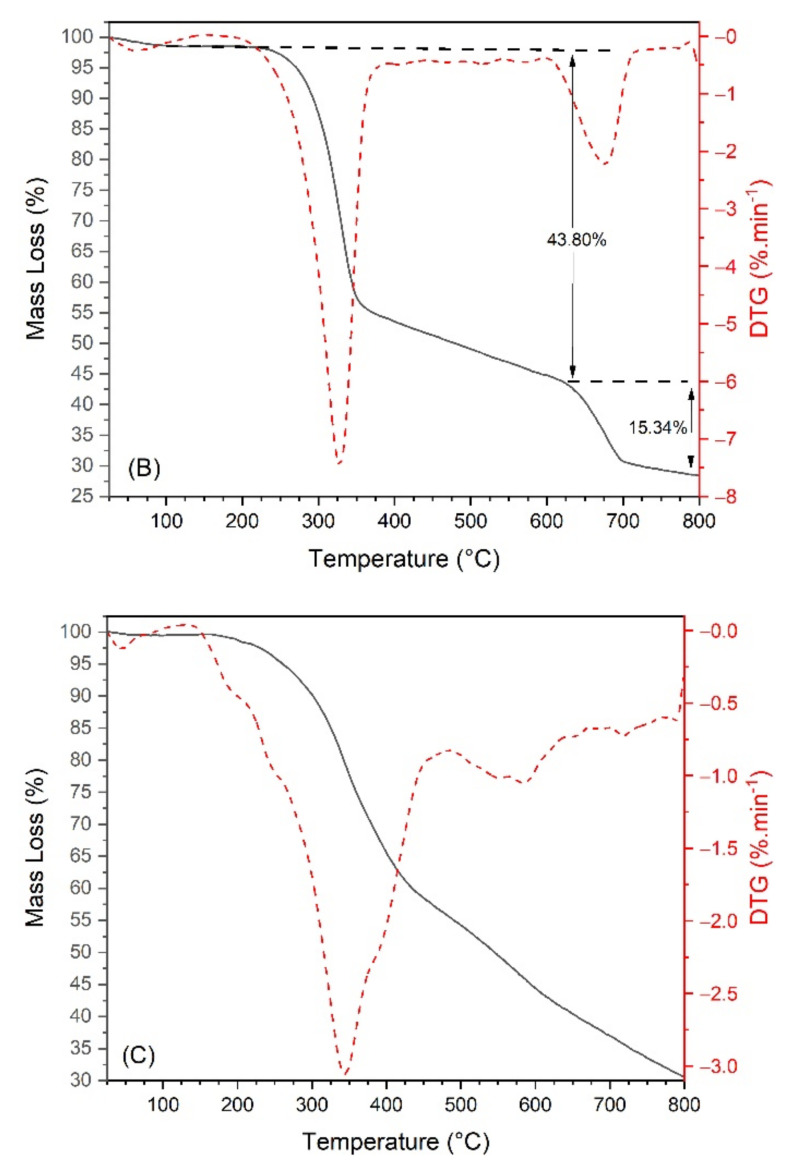
Thermogravimetric curves of (**A**) polylactic acid (PLA), (**B**) sludge fiber waste (SFW), and (**C**) kraft lignin powder (KLP).

The thermal behavior of the PLA, SFW, KLP, and biocomposites was studied by thermogravimetry. From Figure 1A, PLA shows an onset temperature of 332 °C and only one degradation phase, resulting in greater thermal stability than the SFW and KLP. The SFW presents two degradation phases (Figure 1B), the first one with the maximum mass loss at 330 °C due to cellulose and hemicellulose pyrolysis [2,32] and the second one at a maximum mass loss rate of 679 °C related to the decomposition of CaCO_3_ [33,34]. From the second stage of mass loss present on the SFW thermogravimetric curve between 620 and 800 °C, corresponding to the decomposition of CaCO_3_, it was possible to estimate the amount of this mineral present in the SFW [35,36]. The mass difference was 15.34% due to the release of CO_2_ from CaCO_3_, thus, stoichiometrically, the estimated amount of CaCO_3_ present in the SFW was 34.9%. The sum of amounts of CaCO_3_ and the first stage of degradation of 43.8% (attributed to cellulose and organic compound decomposition) does not correspond to 100% of the total. The difference of 21.3% was due to another inorganic compound present in the SFW (quartz), which is thermally stable up to the temperature of 800 °C.

The higher KLP mass loss occurred between 300 and 500 °C (Figure 1C) and is related to the fragmentation of inter-unit linkage and the decomposition of products having a phenolic hydroxyl group [12,37]. KLP reached a maximum mass loss rate at 344 °C, presenting a higher char residue content at 800 °C (30.67%) among the raw materials. The high char residue is correlated to the carbon yield, which is not entirely decomposed in an N_2_ atmosphere by the absence of O_2_ and no oxidation [38].

**Figure 2:**
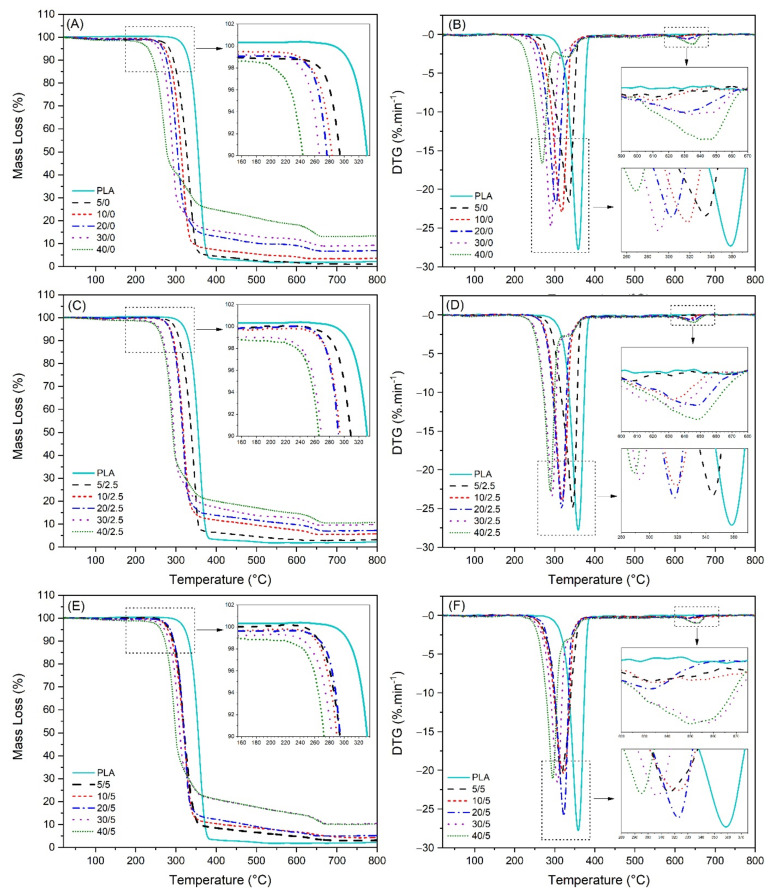
Thermogravimetric curves of biocomposites: (**A**) TG curves for compositions with no KLP, (**B**) DTG curves for compositions with no KLP, (**C**) TG curves for compositions with 2.5% KLP, (**D**) DTG curves for compositions with 2.5% KLP, (**E**) TG curves for compositions with 5% KLP, (**F**) DTG curves for compositions with 5% KLP.

The thermogravimetric curves of the biocomposites show lower thermal stability than PLA (Figure 2). Three main mass losses could be observed, mainly in the biocomposites with a higher amount (40%) of incorporated SFW. The first peak was between 200 and 280 °C, indicating the cellulose decomposition, and it is represented in the DTG curve as the sharpest peak. The second one between 280 and 370 °C is related to PLA decomposition and could be visualized on the DTG curve as a shoulder along with the first peak. The final loss could be observed at the end of the TG curve between 600 and 700 °C, corresponding to CaCO_3_ decomposition present in the SFW appearing at the end of the DTG curve as the last pronounced peak.

Table 2 shows the main temperature characteristics of the biocomposites and the thermal stability could be analyzed by T_onset_. As can be seen, there was a clear trend in the biocomposite behavior without KLP incorporation, decreasing the thermal stability and increasing the char residue according to the proportion of SFW. This might be due to the decrease of the relative molecular weight of biocomposites with the incorporation of SFW in the PLA matrix. As can be previously observed in Figure 1B, the cellulose fibers contained in the SFW were thermally less stable than PLA, which may have resulted in a negative effect on the biocomposites. Even with the incorporation of low proportions of SFW (5% and 10%), the stability decreased by at least 10%. This trend was minimized with the addition of 2.5% of KLP, increasing the stability of the biocomposite composed by 5/2.5 at 4.27% and almost matched the stability of the biocomposites composed by 0/5, 20/5, 30/5 and 40/5. The addition of 2.5% KLP resulted in the increase of char residue in a general way; however, when 5% of KLP was added, there was the tendency to reduce the residue slightly. Indeed, the addition of lignin tended to increase the total char residue as previous results reported [25,39,40]. In that case, the heterogeneous dispersion of CaCO_3_ present in the SFW could have influenced mainly biocomposites with high amounts of incorporation, since this one may be present in a greater proportion with respect to the fibers in the SFW. In contrast, the addition of 5% KLP improved the thermal stability of biocomposites except for the one containing 5% SFW. The addition of 5% KLP, in fact, brought the stability temperatures of the biocomposites closer each other, being able to maintain also similar amounts of residue. Lignin is an environmentally friendly flame-retardant additive that improves thermal oxidation stability due to the abundance of aromatic structures in its composition, yielding high carbon residues after combustion [41,42,43]. In this study, the addition of KLP as a stabilizer was able to increase or maintain the decomposition temperatures of the biocomposites.

### 3.2. Water Absorption

**Figure 3:**
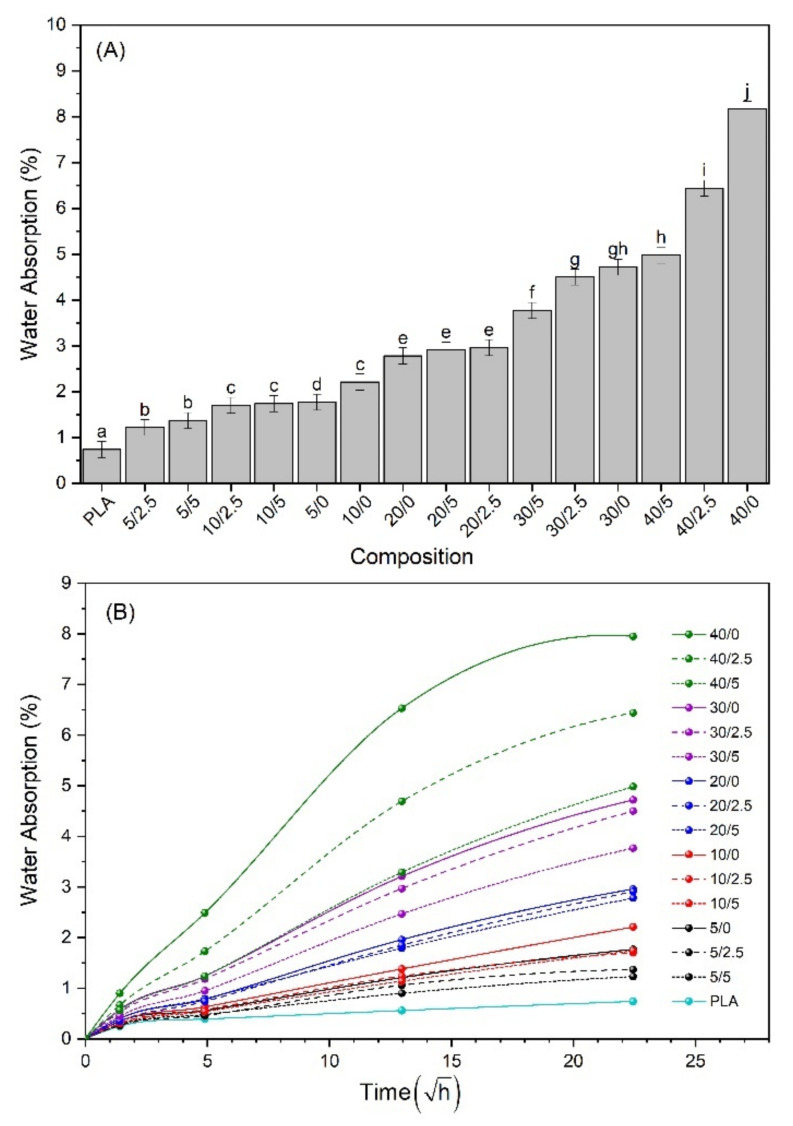
(**A**) Water absorption after saturation; (**B**) water absorption as a function of square root of the time.

Water absorption (WA) of the biocomposites is shown in Figure 3, as well as the results of the multivariate analysis. The WA increased continuously with the time of immersion and reached equilibrium after 504 h. The incorporation of SFW in PLA increased the WA of biocomposites proportionally to the SFW ratio (Figure 3A). The hydrophilic characteristics of cellulose and hemicellulose contained in SFW explain this phenomenon. In fact, as already mentioned in the literature, when the biocomposite is consolidated with high-fiber volume fractions, it results in the formation of clusters of connected material that are not completely isolated by the matrix that then remain on the surface, therefore becoming a point of percolation of water into the composite, thus providing continuous conduction paths of water by fibers [44,45].

Swelling of cellulose when exposed to water results in microcracking of the matrix, contributing to extended water paths and then more water penetration into the biocomposites, resulting in de-bonding between the fiber and the matrix [46]. However, the addition of KLP significantly reduced the WA up to 30% of SFW and showed an even more pronounced effect with 40% of SFW. The incorporation of KLP decreases the WA during the time (Figure 3B). KLP acts as a stabilizer or countermeasure effect in the gain of water caused by the addition of SFW. This effect on the WA, well known in lignocellulosic materials, such as wood cell walls, is due to the increase of hydrophobic aromatic rings of lignin such that these polar ends would interact favorably with the surfaces of cellulosic fibers, partially preventing WA in the composite [47,48,49].

### 3.3. Mechanical Properties

**Figure 4:**
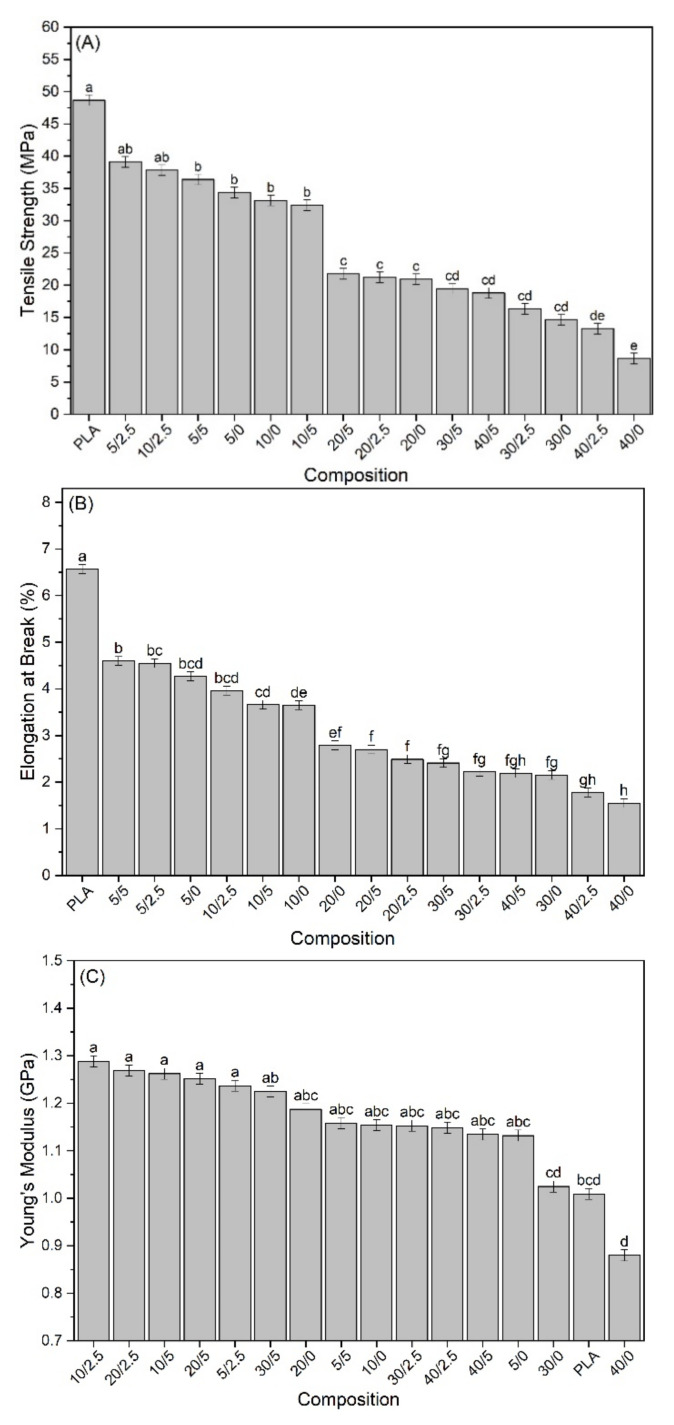
(**A**) Tensile strength; (**B**) Elongation at break, and (**C**) Young’s modulus of biocomposites.

The tensile properties of the biocomposites and their multivariate analysis results are shown in Figure 4. It is observed that the tensile strength of the biocomposites decreased with the incorporation of SFW (Figure 4A). In this case, it can be stated that SFW does not have sufficient properties to satisfactorily improve the strength of the composites or the adhesion between SFW and PLA, and was not able to transfer the possible reinforcement properties of SFW to the biocomposites. However, the addition of 2.5% of KLP in the biocomposites with a low proportion of SFW (5% and 10%) balanced the strength, showing no significant differences compared with PLA. The increase of 2.5% to 5% lignin in the biocomposites with a low SFW content did not show significant losses in resistance. The great loss in strength began with the incorporation of 20% of SFW, decreasing drastically with 40%. By adding 5% KLP, it was possible to maintain unaltered the resistance of biocomposites containing 20% to 40% SFW.

Similar behavior to the tensile strength is observed in the elongation at break (Figure 4B). The elongation of biocomposites decreased significantly with the incorporation of SFW from low proportions. In that case, the addition of KLP showed no significant improvement or loss of stabilization.

There were no significant changes in the Young’s modulus (YM) of the biocomposites with the incorporation of SFW when compared with PLA (Figure 4C). However, the combination between the addition of 2.5% and 5% of KLP until 20% of SFW showed significantly higher values than PLA. The YM began to decrease slightly in biocomposites with 30% and 40% of SFW, even combined with the addition of KLP. In this case, the amount of KLP was insufficient to efficiently encapsulate the fibers and compensate the decrease caused by the high content of SFW. In addition, high proportions of SFW brought greater amounts of CaCO_3_, which may have a significant effect on the YM, as explained for the tensile strength. Therefore, in order to reach the maximum performance of YM, the incorporation threshold of 20% SFW associated with 2.5% and 5% KLP is suggested.

Modulus of rupture (MOR) shown a similar trend to that observed for tensile strength and elongation at break. Significant changes were not observed in MOR between PLA and the biocomposites with incorporation of 5% and 10% of SFW and the addition of KLP in both amounts. The tendency to reduce the tensile strength, elongation, and MOR with the incorporation of SFW in PLA composites was also observed in [50].

Significant losses in these properties could be associated with the weak interfacial adhesion between the hydrophilic cellulose fibers contained in the SFW and PLA matrix [39]. In addition, the high content of mineral compounds, mainly CaCO_3_ in SFW, may have contributed to reducing these properties. Betancourt and Cree (2017) observed similar behavior in their previous study with different loadings of CaCO_3_ in PLA composites and attributed these results to the agglomeration of high amounts of filler, leading to reduced bond strength between particles and an overall poorer dispersion. The weak bonding between CaCO_3_ particles and PLA is due to the aggregates acting as failure-initiation producing sites where micro-cracks can initiate by stress concentrations [8,51,52].

**Figure 5:**
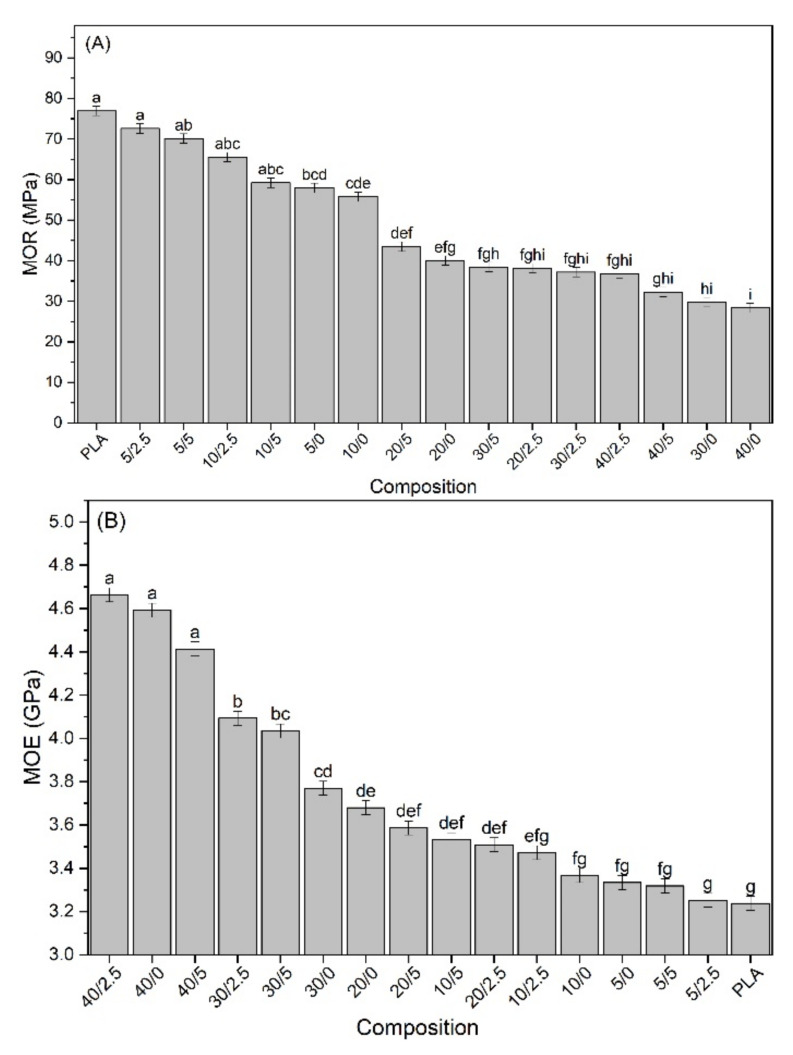
(**A**) Modulus of rupture and (**B**) modulus of elasticity of biocomposites.

The positive effect of KLP was not observed on the modulus of elasticity (MOE) results (Figure 5B). In that case, the tensile modulus increased with the increase in the SFW content. Despite that, this increase was significant only with the incorporation of 20% or more of SFW when compared with PLA. In a general way, the biocomposites composed of 40% SFW presented significantly better results. It is known that the addition of fibrous fillers in a polymeric matrix generally increases the MOE of composites due to stress transfers from the PLA matrix to the stiffer fibers [53]. On this basis, the incorporation of SFW improved the stiffness of the biocomposites according to the proportion and significantly above 20%, regardless of the amount of KLP added.

In a general way, the results were inconclusive with the addition of SFW in biocomposites behaving only as a filler (due to high inorganic content) and showing poor adhesion. However, the addition of KLP shows evidence that it has the potential for promoting adhesion between cellulose content in SFW and PLA, besides enhancing hydrophobicity and having dispersive, lubricant, and thermal stability effects.

### 3.4. SEM Analysis

**Figure 6:**
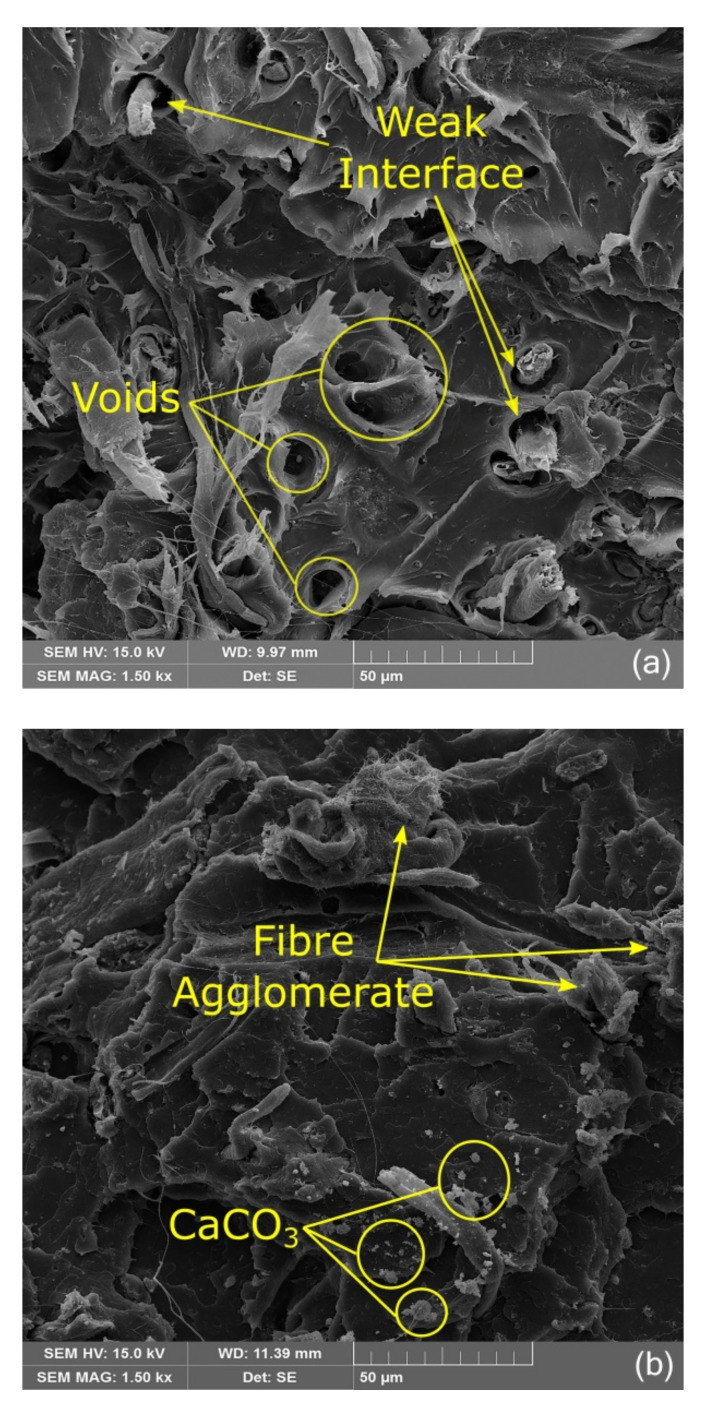

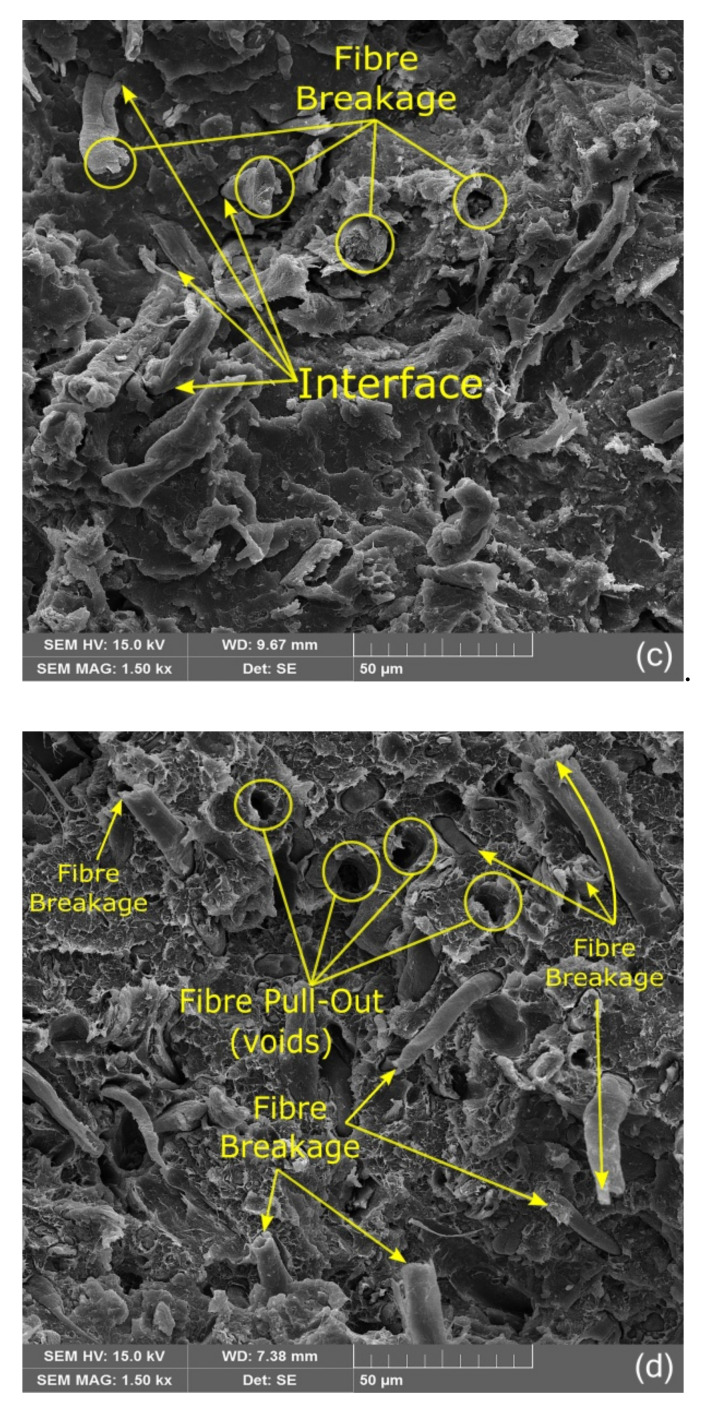
SEM micrographs of surfaces at the tensile rupture point: Evidence observed in (**a**) composition 10/0; (**b**) composition 30/0; (**c**) composition 20/2.5; (**d**) composition 40/5.

According to SEM micrographs it could be observed that the biocomposites produced without the addition of KLP showed low or the absence of adhesion between the fibers of SFW and the PLA, characterized by typical fiber pull-out (Figure 6a), which indicates a lack of interface (marked by arrows) due to the hydrophilic nature of SFW and the hydrophobic nature of the matrix [54].

It is possible to identify voids (marked by circles), which indicate the presence of fibers that were not strength ruptured but pulled out from the matrix. This behavior is easier to observe at low SFW ratio composites (5% and 10%), which could be explained in two different ways. In low proportions of SFW, there is a smaller amount of fibers that are agglomerated or awfully close together, which allows for better viewing.

The other explanation is because, in high proportions of SFW, the fibers occupy a larger volume of the composite and the matrix becomes brittle. When the tensile load is applied, the matrix surrounding the fibers shows a larger surface of contact, allowing for a better load transfer to the fibers (or even a local fiber network) that are then ruptured consecutively. In both ways, the transfer of stress from the matrix to the fibers was not completely efficient. The increase of proportion of SFW in the biocomposites without the addition of KLP increased consequently the number of regions with fiber agglomerates (Figure 6b marked by arrows) and the amount of CaCO_3_ (Figure 6b marked by circles) related to an ineffective dispersion of SFW in matrix. All these factors contribute to generating composites with inferior properties to those with KLP addition.

The addition of KLP in the biocomposites reduced part of the problems cited previously. The interface between fibers and PLA was improved with addition of 2.5% and 5% of KLP, and it could be visualized by better contact or a decrease of voids between fibers and matrix and the higher amount of broken fibers (Figure 6c). In addition, broken fibers could be detected as a result of stress absorption from the matrix. Indeed, the addition of KLP reduced the polarity at the interface, increasing interfacial adhesion, despite in some cases where the high amount of lignin could coat fibers and then decrease the physical anchoring between the fibers and matrix [26]. This behavior was more pronounced in biocomposites with the incorporation of low proportions of SFW, since higher amounts of SFW tended to generate agglomerates of fibers, as well as the same happening with high amounts of KLP.

This analysis confirms the best mechanical results were obtained by biocomposites with low proportions of SFW and the addition of KLP. The benefits of KLP addition could be observed to some extent, since the greater the amount of SFW and KLP incorporated (40%/5%), the lower the capacity of the polymer to cover all the fillers efficiently. In addition, due to this study using the maximum addition of 5% KLP, there might not have been enough KLP available to fully cover all the fibers of SFW. Thus, some regions displaying good adhesion presented broken fibers (Figure 6d marked by arrows), but in other ones, weak adhesion presented voids (Figure 6d marked by circles) and consequently prejudiced the mechanical properties of the material.

## 4. Conclusions

It was concluded that the incorporation of SFW decrease the thermal stability of biocomposites; however, the addition of KLP had the opposite effect, contributing to stabilize and improve the lost resistance to heat.

The CaCO_3_ content in SFW significantly affected the reinforcing capacity of SFW, mainly with SFW ratio above 10%.

The water absorption increased significantly and proportionally with the incorporated amount of SFW. The addition of KLP slightly prevented water absorption, but not significantly.

SEM micrographs revealed low or no adhesion between the fibers from SFW and the PLA in compositions without KLP added. In addition, KLP was able to improve the interfacial adhesion only in compositions with low SFW ratio. KLP also improved the performance of the biocomposite by acting as a flame retardant, dispersion or coupling agent, and reducing water absorption.

It was also concluded that SFW and KLP have great potential as a filler for PLA biocomposites, reducing costs due to the lower PLA content in the material, and offer a better destination for waste and by-products of paper mills, allowing them to produce a material with higher value added as lightweight and non-structural products, such as plants tubes and pots, and even interior parts for the automobile industry. Therefore, to better target these biocomposites in relation to their application, it is necessary to study and define the optimal ratio between SFW, KLP, and PLA.

## Figures and Tables

**Table 1:** Experimental design.

Proportion (wt.%)		
PLA	SFW	KLP
100	0	0
95	5	0
90	10	0
80	20	0
70	30	0
60	40	0
92.5	5	2.5
87.5	10	2.5
77.5	20	2.5
67.5	30	2.5
57.5	40	2.5
90	5	5
85	10	5
75	20	5
65	30	5
55	40	5

**Table 2:** Some thermal characteristics of PLA, SFW, KLP, and biocomposites.

Composition	T_onset_ (°C)	T_1max_ (°C)	T_2max_ (°C)	CR_T1max_ (%)	CR_T2max_ (%)	CR_800_ (%)
PLA	332	359	-	39.38	-	2.18
SFW	290	330	679	69.46	34.51	28.46
KLP	269	344	582	79.52	46.29	30.67
5/0	298	335	-	32.91	-	1.20
10/0	287	319	608	36.76	4.01	3.60
20/0	278	302	627	53.88	8.03	6.98
30/0	270	291	634	51.66	10.31	9.23
40/0	241	269	640	62.82	15.19	13.40
5/2.5	313	346	-	31.24	-	3.22
10/2.5	295	319	632	48.78	6.35	5.77
20/2.5	293	318	647	46.60	7.80	7.27
30/2.5	272	293	632	55.43	10.82	9.78
40/2.5	270	289	649	57.84	11.78	10.75
5/5	294	316	-	59.27	-	3.16
10/5	295	322	636	44.76	6.01	4.34
20/5	298	323	632	43.05	5.66	5.21
30/5	284	307	659	56.25	11.45	10.45
40/5	275	295	649	59.86	12.05	10.27

T_1max_ = maximum first peak temperature; T_2max_ = maximum second peak temperature; CR_T1max_ = charred residue at T_1max_; CR_T2max_ = charred residue at T_2max_; CR_800_ = charred residue at 800 °C.

## Data Availability

The data presented in this study are available on request from the corresponding author.
